# Epigenetic Dysregulation Observed in Monosomy Blastocysts Further Compromises Developmental Potential

**DOI:** 10.1371/journal.pone.0156980

**Published:** 2016-06-07

**Authors:** Michelle M. Denomme, Blair R. McCallie, Jason C. Parks, William B. Schoolcraft, Mandy G. Katz-Jaffe

**Affiliations:** 1 Fertility Labs of Colorado, Lone Tree, Colorado, United States of America; 2 Colorado Center for Reproductive Medicine, Lone Tree, Colorado, United States of America; University of Bonn, Institute of Experimental Hematology and Transfusion Medicine, GERMANY

## Abstract

Epigenetic mechanisms such as DNA methylation regulate genomic imprinting and account for the distinct non-equivalence of the parental genomes in the embryo. Chromosomal aneuploidy, a major cause of infertility, distorts this highly regulated disparity by the presence or absence of chromosomes. The implantation potential of monosomy embryos is negligible compared to their trisomy counterparts, yet the cause for this is unknown. This study investigated the impact of chromosomal aneuploidy on strict epigenetically regulated domains, specifically imprinting control regions present on aneuploid chromosomes. Donated cryopreserved human IVF blastocysts of transferable quality, including trisomy 15, trisomy 11, monosomy 15, monosomy 11, and donor oocyte control blastocysts were examined individually for DNA methylation profiles by bisulfite mutagenesis and sequencing analysis of two maternally methylated imprinting control regions (ICRs), *SNRPN* (15q11.2) and *KCNQ1OT1* (11p15.5), and one paternally methylated imprinting control region, *H19* (11p15.5). Imprinted genes within the regions were also evaluated for transcript abundance by RT-qPCR. Overall, statistically significant hypermethylated and hypomethylated ICRs were found in both the trisomy and monosomy blastocysts compared to controls, restricted only to the chromosome affected by the aneuploidy. Increased expression was observed for maternally-expressed imprinted genes in trisomy blastocysts, while a decreased expression was observed for both maternally- and paternally-expressed imprinted genes in monosomy blastocysts. This epigenetic dysregulation and altered monoallelic expression observed at imprinting control regions in aneuploid IVF embryos supports euploid embryo transfer during infertility treatments, and may specifically highlight an explanation for the compromised implantation potential in monosomy embryos.

## Introduction

Embryonic chromosomal aneuploidy is a major cause of human infertility, and likely contributes to most failed conceptions, both natural and IVF. As the most common chromosome abnormality in human reproduction, it is the leading source of miscarriages, stillbirths, and congenital birth defects [1], as well as the largest underlying basis for embryo arrest and implantation failure [2]. Over half of early human IVF embryos are estimated to be affected by whole chromosome imbalances. While it is difficult to definitively decipher the precise frequencies of meiotic and mitotic origins, it is known that the vast majority are a result of maternal meiotic errors in the oocyte, followed distantly by mitotic errors post-fertilization, having paternal meiotic errors in the sperm occurring at the lowest frequency [3, 4].

Aneuploidy can affect any of the twenty-two autosome pairs and the two sex chromosomes. However, trisomy and monosomy errors compromise embryonic development by different means. Trisomy errors involve the gain of a third additional chromosome. Many of the smaller size trisomies will successfully implant, but contribute to pregnancy loss, and only a handful can reach live birth including the widely characterized Down Syndrome (Trisomy 21). Monosomy errors involve the loss of one chromosome from a pair. These embryos predominately do not implant, and rarely reach clinical pregnancy, having Turner Syndrome (XO) the only full monosomy that has the potential to develop to term. This significant disparity reported between pre- and post-implantation frequencies for monosomy embryos has recently been tallied, with 45.7% of preimplantation monosomies in blastocysts reduced to 6.3% of post-implantation monosomies in spontaneous abortuses [5]. The cause for this compromised implantation potential for monosomy embryos compared to their trisomy counterparts is unknown. An increasing number of aneuploidy studies have proposed a relationship between chromosome imbalance and epigenetics, though cause and consequence are still undetermined [6–12].

Epigenetics describes the chromatin modifications that alter the accessibility of genes and regulate gene expression [13]. These chromatin changes allow cells to maintain differential expression despite containing the same genomic material [14]. Gametogenesis and early embryogenesis are critical stages in which genome-wide epigenetic transitions required for early mammalian development are orchestrated [15].

The epigenetic phenomenon known as genomic imprinting restricts gene expression in the embryo to one of the two parental contributions. Disruption to this highly regulated process can lead to developmental and neurological disorders such as Beckwith-Wiedemann Syndrome (BWS), Angelman Syndrome (AS) and Prader-Willi Syndrome (PWS), which are characterized by genetic and epigenetic errors at the *KCNQ1OT1* (BWS), *H19* (BWS) and *SNRPN* (AS and PWS) imprinting control regions (ICRs) [16–22]. Epigenetic mechanisms, including DNA methylation, are criticial for maintaining genomic imprinting regulation. The hypothesis is that chromosomal aneuploidy distorts this highly regulated disparity, with some evidence observed in neoplasia [8, 23].

Our previous investigation of epigenetic modifiers uncovered a hypomethylated state and reduced gene expression limited to the monosomy chromosome [24]. To further analyze the epigenetic effects of aneuploidy, in this study we compared the imprinted DNA methylation profiles and imprinted gene expression levels of three ICRs, *SNRPN*, *H19* and *KCNQ1OT1*, in individual aneuploid blastocysts. Consistent with our expectations, trisomy and monosomy blastocysts had both hyper- and hypomethylated imprinted DNA regions and altered imprinted gene expression when compared to controls. Our results indicate that the dysregulation of genomic imprinting in aneuploid blastocysts, specifically the altered imprinted methylation leading to decreased gene expression observed in monosomy embryos, may contribute to their reduced implantation potential and support euploid embryo transfer following comprehensive chromosome screening in IVF cycles.

## Materials and Methods

### Surplus cryopreserved human blastocysts

One hundred and thirty five patients who had undergone fertility treatments at the Colorado Center for Reproductive Medicine (CCRM) donated a total of 170 cryopreserved human blastocysts. All blastocysts were viable and morphologically graded as high transferrable quality ≥3BB on Day 5 of embryonic development [25]. Comprehensive chromosome screening (CCS) was performed by our in-house genetics laboratory at CCRM as previously described [26]. Trisomy 15 (n = 30), monosomy 15 (n = 30), trisomy 11 (n = 30), monosomy 11 (n = 35), and donor oocyte control blastocysts (n = 45) were analyzed. Blastocysts were divided between two experimental models, either imprinted DNA methylation analysis or imprinted gene expression analysis, as described in Table 1.

**Table 1:** Summary of blastocysts divided among experimental procedures.

Experiment	Embryos	Number of Blastocysts	Number of Patients	Mean Maternal Age	Mean Paternal Age
	Controls	35	13	Donor, <33	39.7 ± 5.2
Imprinted	Trisomy 15	20	20	39.5 ± 3.8	40.0 ± 6.5
DNA	Trisomy 11	20	20	38.7 ± 4.0	39.9 ± 4.4
Methylation	Monosomy 15	20	20	38.3 ± 4.4	37.2 ± 4.3
	Monosomy 11	25	25	39.0 ± 3.0	39.7 ± 4.6
	Controls	10	2	Donor, <33	39.8 ± 1.5
Imprinted	Trisomy 15	10	10	40.5 ± 2.1	41.8 ± 5.5
Gene	Trisomy 11	10	10	37.1 ± 4.4	39.7 ± 6.5
Expression	Monosomy 15	10	10	41.2 ± 2.3	44.8 ± 5.6
	Monosomy 11	10	10	40.9 ± 1.2	42.8 ± 3.2

### Ethics statement

Ethics approval was obtained from our associated HCA-HealthONE Institutional Review Board and Western Institutional Review Board. All patients provided informed written consent for their blastocysts to be used in this study and gave permission for researchers to access medical records to obtain embryology and CCS results.

### Imprinted DNA methylation analysis by bisulfite mutagenesis

Bisulfite mutagenesis and clonal sequencing of individual human blastocysts was performed as previously described [27, 28]. Briefly, warmed blastocysts were embedded in a 2:1 low melting point agarose and lysis mixture and incubated in SDS lysis buffer overnight in a 50°C waterbath. The following day, the samples were immediately processed for bisulfite mutagenesis or frozen at (-20°C) for a maximum of 5 days. To process, samples were first incubated at 90–95°C for 2.5 minutes and were then denatured for 15 minutes at 37°C in 1 mL of 0.1M NaOH. Bisulfite conversion occurred in 500 μL of 2.5M bisulfite solution under mineral oil in a 50°C waterbath for 3.5 hours. Desulfonation of samples took place at 37°C for 15 minutes in 1 mL of 0.3M NaOH, and the samples were then washed twice in 1 mL of TE buffer and twice in 1 mL of H_2_O with shaking.

For the first round of nested PCR amplification, the agarose bead was added directly to a Hot Start Ready-To-Go (RTG) (GE Healthcare) PCR bead that contained 0.5 μL of each 10 μM gene-specific outer primer, 1 μL of 240 ng/mL transfer RNA and water up to 15 μL. For the second round of nested PCR amplification, 5 μL of first round product was added to a fresh RTG bead mixed with 19 μL water and 0.5 μL of each 10 μM inner primer. *The SNRPN*, *H19*, and *KCNQ1OT1* PCR bisulfite primers were described previously [27] and can be found in Table 2. Both outer and inner PCR reactions were performed as previously described [27]. Following the second round of PCR amplification, samples were gel extracted, ligated into the pGEM-T EASY vector system (Promega), and transformed into Z-competent DH5α *Escherichia coli* cells (Zymo Research). Following colony PCR amplification, 30 μL of individual clone samples were sent for sequencing at Bio Basic Inc. (Markham, ON, Canada).

**Table 2:** PCR primers and parameters for bisulfite mutagenesis.

*Locus*	*Gene*	*Primer Name*	*Primer Sequence (5’-3’)*	*Size (bp)*	*CpG (#)*	*SNPs*	*REF*
*15q11*.*2*	***SNRPN***	*SNRPN-OF*	TAGTGTTGTGGGGTTTTAGGG	*371*	*24*	*Rs220029*	[27]
	*(U41384)*	*SNRPN-IF*	AGGGAGGGAGTTGGGATTT			*G (84*.*8%) /*	
		*SNRPN-IR*	CACAACAACAAACCTCTAAACATTC			*A (15*.*2%)*	
		*SNRPN-OR*	TACCCACCTCCACCCATATC				
*11p15*.*5*	***H19***	*H19-OF*	AATAATGAGGTGTTTTAGTTTTATGGATG	*170*	*14*	*Rs2071094*	[27, 29]
	*(Af087017)*	*H19-IF*	TTGGTTGTAGTTGTGGAAT			*C (66*.*4%) /*	
		*H19-IR*	ACTCCTATAAATATCCTATTCCCAAATAACCCC			*A (33*.*6%)*	
		*H19-OR*	ACTTAAATCCCAAACCATAACACTAAAAC				
*11p15*.*5*	***KCNQ1OT1***	*KCNQ1OT1-OF*	TGTTTTTGTAGTTTATATGGAAGGGTTAA	*265*	*22*	*Rs56134313*	[29, 30]
	*(U90095)*	*KCNQ1OT1-IF*	GTTAGGGAAGTTTTAGGGTGTGAAT			*G (94*.*0%) /*	
		*KCNQ1OT1-IR*	AAACATACCAAACCACCCACCTAACAAA			*A (6*.*0%)*	
		*KCNQ1OT1-OR*	CTCACCCCTAAAAACTTAAAACCTC				

OF = outer forward primer, IF = inner forward primer, IR = inner reverse primer, OR = outer reverse primer

94°C for 10 min, 55 cycles of (94°C for 15 sec, 56°C for 20 sec, 72°C for 20 sec), 72°C for 10 min. [27]

Approximately 25–35 clones were sequenced per blastocyst per gene. Methylation patterns were determined using two online software programs (BISMA and QUMA). Identical clones were considered to be representative of one individual DNA strand, and thus were included only once in each blastocyst DNA methylation figure. Total DNA methylation for each gene was calculated as a percentage of the total number of methylated CpGs divided by the total number of CpG dinucleotides. When present, total DNA methylation for each single nucleotide polymorphism (SNP) was calculated separately. For simplicity of analysis, any monosomy blastocyst containing more than one parental SNP within a gene was discarded from the study. Student’s t-test was used to examine significance for methylation between aneuploid and control blastocysts. A p-value of <0.05 was considered to be statistically significant.

### Relative imprinted expression analysis

Total RNA was isolated from individual whole blastocysts for analysis of gene transcript abundance as previously described [31]. Briefly, using the PicoPure RNA Isolation Kit (Molecular Devices), blastocysts were lysed, treated with RNase-free DNase I (Qiagen), washed twice, and eluted in 20 μL elution solution. To generate cDNA template for real-time PCR, reverse transcription was performed using the High Capacity Reverse Transcription cDNA kit (Applied Biosystems).

Quantitative real-time PCR was performed using the ABI 7300 Real Time PCR System with the Power SYBR Green PCR Master Mix (Applied Biosystems). The quantification of two genes for trisomy 15 and monosomy 15, *SNRPN* and *UBE3A*, and three genes for trisomy 11 and monosomy 11, *H19*, *KCNQ1OT1* and *CDKN1C* were calculated relative to the housekeeping gene, *PPIA* (7p13), which displayed comparatively constant levels of transcription in every sample, and compared to donor control blastocysts. Expression primers and PCR parameters are outlined in Table 3, all primers were designed in-house except for *H19* [32]. The PCR reaction efficiency recorded R^2^ values ≥ 0.95 and the correlation coefficient was calculated to be >0.99. Statistical analysis was performed using the Relative Expression Software Tool (REST-2009) (Qiagen). REST software determines an expression ratio that is tested for significance by a Pair Wise Fixed Reallocation Randomization Test [33]. Samples with p<0.05 were considered statistically significant.

**Table 3:** Expression primers and PCR parameters.

*Locus*	*Gene*	*Primer Name*	*Primer Sequence (5’-3’)*	*Size (bp)*
*15q11*.*2*	***SNRPN***	*SNRPN-F*	GCCATATTGGAGTAGCGAGGAA	84
	*(*NM_003097.3)	*SNRPN-R*	CAATGCAAGCTGGGCAGAA	
	***UBE3A***	*UBE3A-F*	CAAAAATGGCCCAGACACAGA	119
	*(*NM_000462.3)	*UBE3A-R*	ACGTGATGGCCTTCAACAATC	
*11p15*.*5*	***H19***	*H19-F*	GGAGTGAATGAGCTCTCAGG	308
	*(*NR_002196.2)	*H19-R*	CTAAGGTGTTCAGGAAGGCC	
	***KCNQ1OT1***	*KCNQ1OT1-F*	AAAAGCTCCCAACGAGGAA	64
	*(*NR_002728.3)	*KCNQ1OT1-R*	GGCTCTTGGCTGGGTACA	
	***CDKN1C***	*CDKN1C-F*	GGCCTCTGATCTCCGATTTC	67
	*(*NM_000076.2)	*CDKN1C-F*	ACATCGCCCGACGACTTC	
*7p13*	***PPIA***	*PPIA-F*	GCTTTGGGTCCAGGAATGG	59
	*(*NM_001300981.1)	*PPIA-R*	TTGTCCACAGTCAGCAATGG	

F = forward primer, R = reverse primer.

95°c for 10 min, 40 cycles of (95°C for 15 sec, 60°C for 1 min), 95°C for 15 sec, 60°C for 1 min, 95°C for 15 sec, 60°C for 15 sec

## Results

### Blastocysts and imprinting regions

A total of 170 cryopreserved blastocysts were donated with IRB and patient consent, 45 were donor oocyte control blastocysts, while the remaining 125 were aneuploid blastocysts divided between trisomy 15, trisomy 11, monosomy 15 and monosomy 11. A summary of the distribution of blastocysts among experimental procedures, along with mean maternal and paternal ages are outlined in Table 1.

Three defined ICRs were selected for investigation; the *SNRPN* ICR, located on human chromosome 15q11.2, and the *H19* and *KCNQ1OT1* ICRs located adjacently on human chromosome 11p15.5. The *SNRPN* and *KCNQ1OT1* ICRs are maternally methylated in the oocyte and paternally expressed from the unmethylated sperm contribution. The *H19* ICR is reversed, paternally methylated in the sperm and maternally expressed from the unmethylated oocyte contribution. A single nucleotide polymorphism (SNP) was present in all three amplified regions, but at different frequencies: *SNRPN* SNP G (84.8%) / A (15.2%) (Rs220029), *H19* SNP C (66.4%) / A (33.6%) (Rs2071094), and *KCNQ1OT1* SNP G (94.7%) / A (6.3%) (Rs56134313) (S1 Table).

### Imprinted DNA methylation in control blastocysts

**Fig 1:**
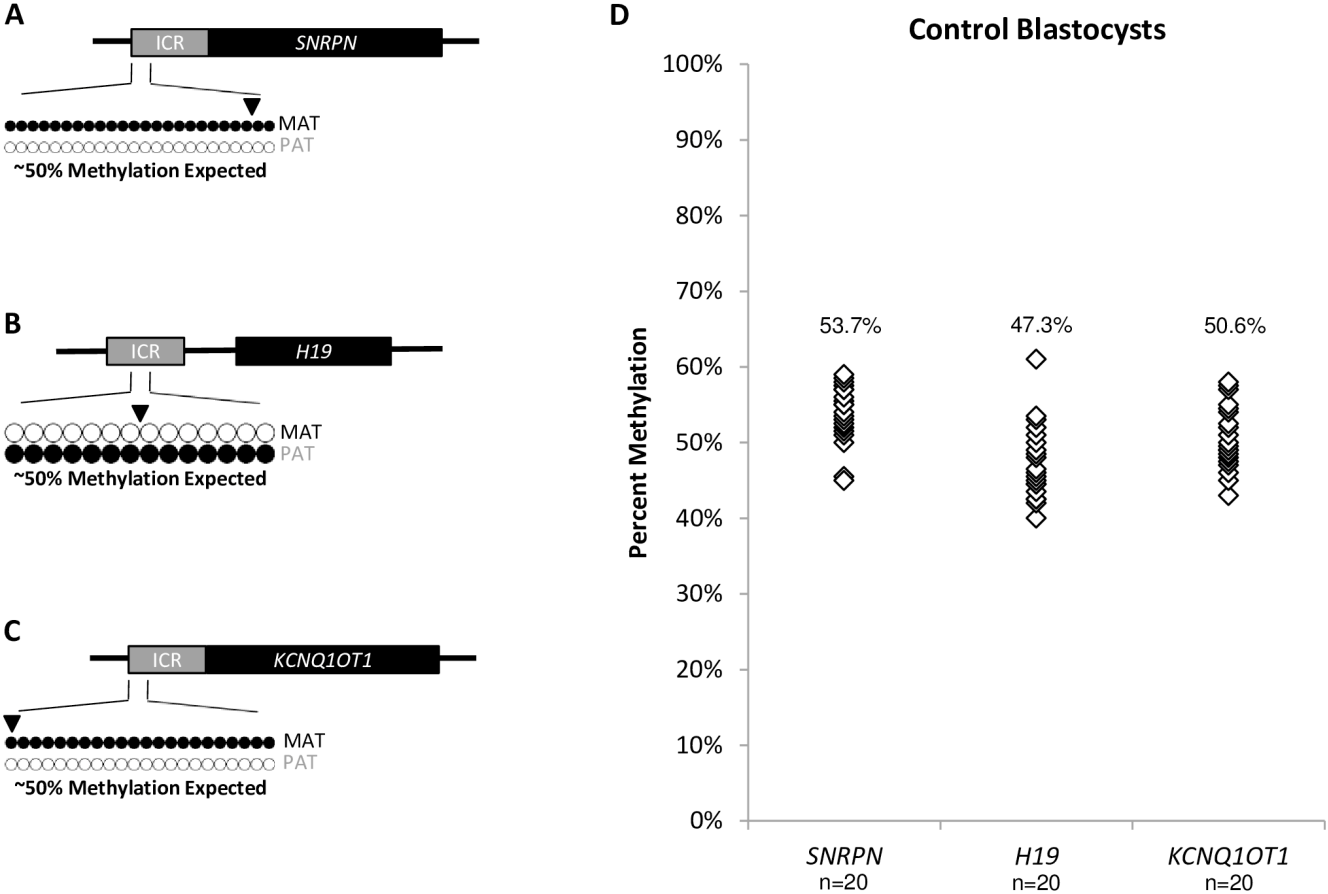
Imprinted DNA methylation profiles of control blastocysts. Black dots represent methylated CpGs, white dots represent unmethylated CpGs. The SNP location is indicated by an arrowhead. “MAT” represents the maternal oocyte-contributed allele; “PAT” represents the paternal sperm-contributed allele. A) Diagram of the *SNRPN* ICR amplified region analyzed on human chromosome 15q11.2 (GenBank: U41384) in control blastocysts, a 371 bp region consisting of 24 assessable CpGs and a G/A SNP (Rs220029). The *SNRPN* ICR is maternally methylated. Approximately 50% methylation is expected, derived from one MAT allele and one PAT allele. B) Diagram of the *H19* ICR amplified region analyzed on human chromosome 11p15.5 (GenBank: AF087017) in control blastocysts, a 170 bp region consisting of 14 assessable CpGs and a A/C SNP (Rs2071094). The *H19* ICR is paternally methylated. Approximately 50% methylation is expected, derived from one MAT allele and one PAT allele. C) Diagram of the *KCNQ1OT1* ICR amplified region analyzed on human chromosome 11p15.5 (GenBank: U90095) in control blastocysts, a 265 bp region consisting of 22 assessable CpGs and a G/A SNP (Rs56134313). The *KCNQ1OT1* ICR is maternally methylated. Approximately 50% methylation is expected, derived from one MAT allele and one PAT allele. D) Summary chart of percent methylation in all control blastocysts at the *SNRPN* (n = 20), *H19* (n = 20), and *KCNQ1OT1* (n = 20) ICRs. Each white diamond represents the methylation percentage for one individual control blastocyst per ICR. Average percent methylation for each cohort is indicated above each gene. Methylation dot diagrams for individual control blastocysts can be found in S1 Fig, and a summary can be found in S2 Table.

Each control blastocyst displayed diploid chromosomes representing one inherited methylated allele and one inherited unmethylated allele at all imprinted regions (Fig 1a–1c). Analysis of the twenty blastocysts examined at *SNRPN* revealed an average methylation of 53.7% ± 3.9%. *H19* had an average methylation of 47.3% ± 5.0%, while *KCNQ1OT1* had 50.6% ± 4.2% average methylation. To summarize, all control blastocysts examined had a DNA methylation profile range that was comparable to the expected fifty percent methylation for diploid chromosomes (Fig 1d, S1 Fig, S2 Table).

### Imprinted DNA methylation in trisomy blastocysts

**Fig 2:**
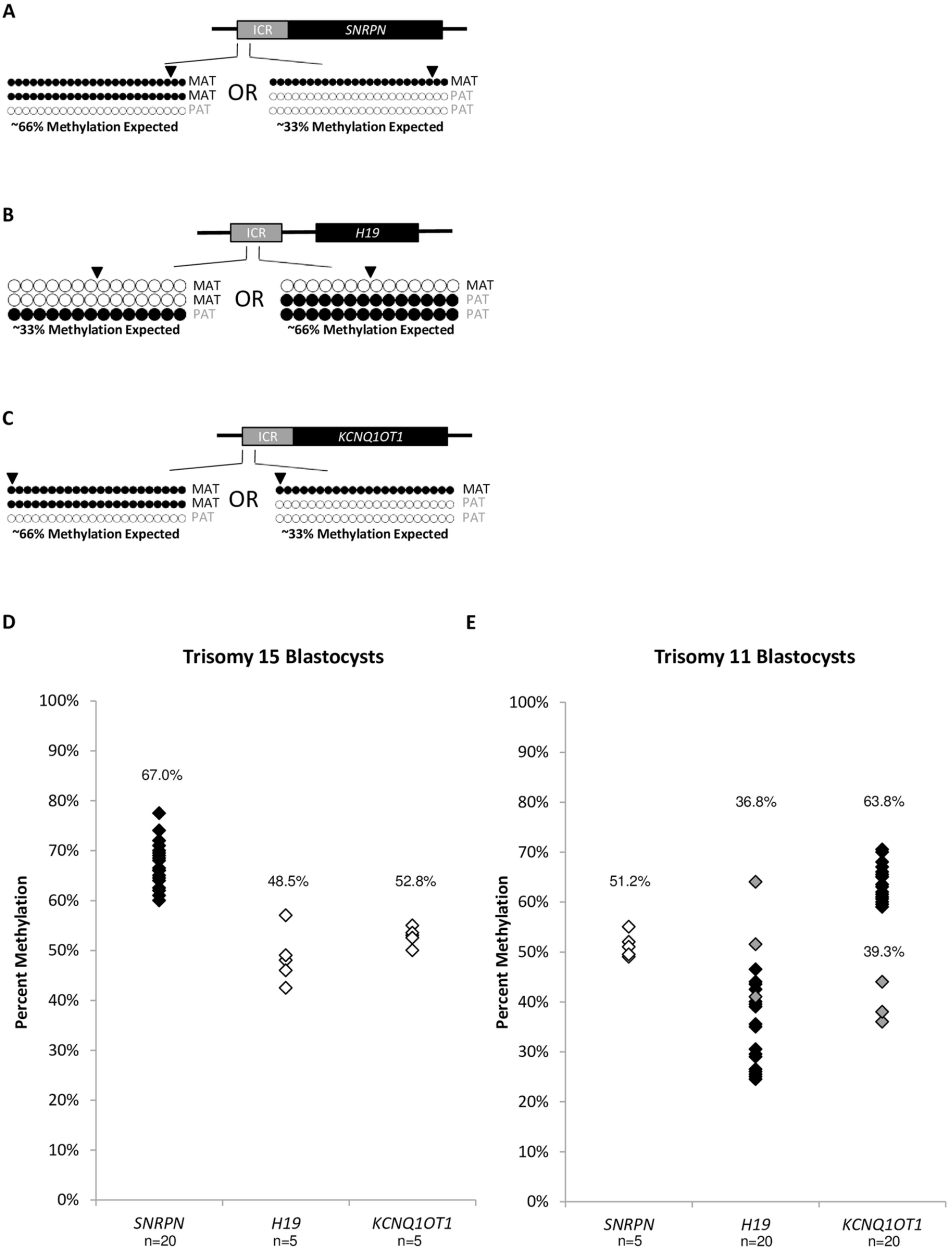
Imprinted DNA methylation profiles of trisomy blastocysts. Black dots represent methylated CpGs, white dots represent unmethylated CpGs. The SNP location is indicated by an arrowhead. “MAT” represents the maternal oocyte-contributed allele; “PAT” represents the paternal sperm-contributed allele. A) Diagram of the *SNRPN* ICR amplified region analyzed in trisomy 15 blastocysts. Approximately 66% methylation is expected with an additional MAT allele. Approximately 33% methylation is expected with an additional PAT allele. B) Diagram of the *H19* ICR amplified region analyzed in trisomy 11 blastocysts. Approximately 33% methylation is expected with an additional MAT allele. Approximately 66% methylation is expected with an additional PAT allele. C) Diagram of the *KCNQ1OT1* ICR amplified region analyzed in trisomy 11 blastocysts. Approximately 66% methylation is expected with an additional MAT allele. Approximately 33% methylation is expected with an additional PAT allele. D) Summary chart of percent methylation in all trisomy 15 blastocysts at the *SNRPN* (n = 20), *H19* (n = 5), and *KCNQ1OT1* (n = 5) ICRs. Black diamonds represent blastocysts with presumable gain of the third chromosome 15 from the oocyte, grey diamonds represent blastocysts with presumable gain of the third chromosome 15 from the sperm. White diamonds represent the methylation percentage for the ICRs on diploid chromosome 11. Average percent methylation for each cohort is indicated. E) Summary chart of percent methylation in all trisomy 11 blastocysts at the *SNRPN* (n = 5), *H19* (n = 20), and *KCNQ1OT1* (n = 20) ICRs. Black diamonds represent blastocysts with presumable gain of the third chromosome 11 from the oocyte, grey diamonds represent blastocysts with presumable gain of the third chromosome 11 from the sperm. White diamonds represent the methylation percentage for the ICR on diploid chromosome 15. Average percent methylation for each cohort is indicated. Methylation dot diagrams for individual trisomy blastocysts and diploid ICRs can be found in S2 and S4 Figs, and a summary can be found in S2 Table.

At imprinted regions, a trisomy chromosome is represented by one inherited methylated allele, one inherited unmethylated allele, and a third inherited allele with unknown origin and subsequent unknown methylation status (Fig 2a–2c). Analysis of the trisomy 15 blastocysts at the *SNRPN* ICR revealed an average methylation of 67.0% ± 4.5% (p<0.001 compared to controls) among the twenty blastocysts, suggesting a gain of the third additional chromosome from the oocyte for all blastocysts examined **(**Fig 2d, S2 Fig, S2 Table). *H19* and *KCNQ1OT1* reside on diploid chromosome 11, and thus were unaffected by the trisomy, having 48.5% ± 5.4% (p = 0.64), and 52.8% ± 1.8% (p = 0.26), respectively, in the five blastocysts examined (Fig 2d, S4 Fig, S2 Table).

Analysis of trisomy 11 blastocysts at *H19* showed 36.8% ± 10.3% (p<0.001) average methylation (Fig 2e, S2 Fig, S2 Table). The DNA methylation range for *H19* was broadened due to the high frequency of both SNP polymorphisms at this locus. In the same trisomy 11 blastocysts, *KCNQ1OT1* had 17/20 blastocysts with 63.8% ± 3.6% (p<0.001) average methylation, reflecting a gain of the third chromosome from the oocyte, and 3/20 blastocysts with 39.3% ± 4.2% (p<0.001) average methylation, indicating a gain of the third chromosome from the sperm (Fig 2e, S2 Fig, S2 Table).

In confirmation of these origins of aneuploidy, both *H19* and *KCNQ1OT1*, located adjacent to each other on chromosome 11, reflect the same gain from the oocyte or sperm in the same individual blastocysts. For example, blastocyst 11T02, 11T08, 11T12 appear to have gained the third chromosome from the sperm at both imprinted loci. *SNRPN* resides on diploid chromosome 15, and was unaffected by the trisomy in all five blastocysts examined, having an average methylation of 51.2% ± 2.5% (p = 0.20), comparable to controls (Fig 2e, S4 Fig, S2 Table).

### Imprinted DNA methylation in monosomy blastocysts

**Fig 3:**
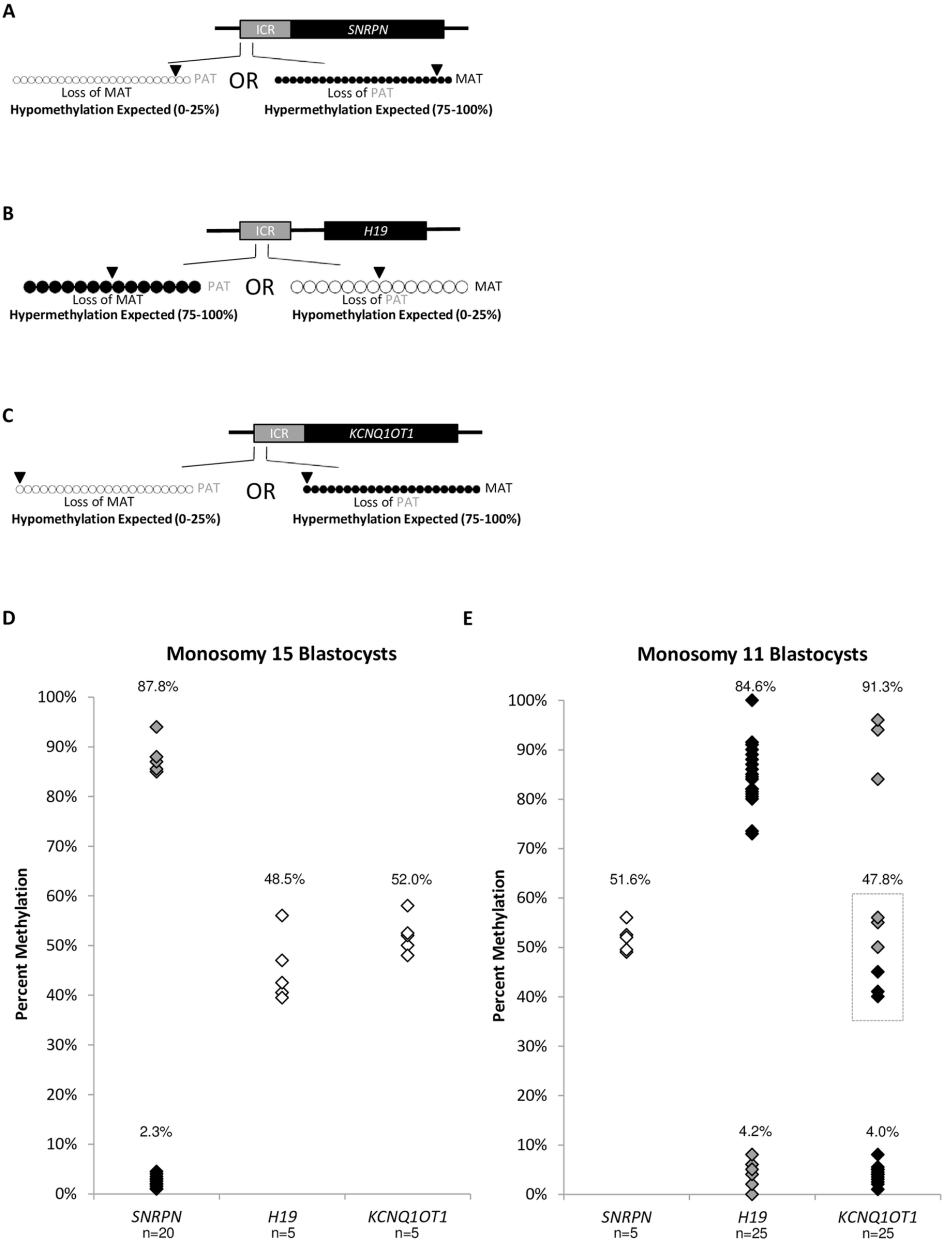
Imprinted DNA methylation profiles of monosomy blastocysts. Black dots represent methylated CpGs, white dots represent unmethylated CpGs. The SNP location is indicated by an arrowhead. “MAT” represents the maternal oocyte-contributed allele; “PAT” represents the paternal sperm-contributed allele. A) Diagram of the *SNRPN* ICR amplified region analyzed in monosomy 15 blastocysts. Hypomethylation (0–25%) is expected with the presence of only the PAT allele. Hypermethylation (75–100%) is expected with the presence of only the MAT allele. B) Diagram of the *H19* ICR amplified region analyzed in monosomy 11 blastocysts. Hypermethylation (75–100%) is expected with the presence of only the PAT allele. Hypomethylation (0–25%) is expected with the presence of only the MAT allele. C) Diagram of the *KCNQ1OT1* ICR amplified region analyzed in monosomy 11 blastocysts. Hypomethylation (0–25%) is expected with the presence of only the PAT allele. Hypermethylation (75–100%) is expected with the presence of only the MAT allele. D) Summary chart of percent methylation in all monosomy 15 blastocysts at the *SNRPN* (n = 20), *H19* (n = 5), and *KCNQ1OT1* (n = 5) ICRs. Black diamonds represent blastocysts with presumable loss of a chromosome 15 from the oocyte, grey diamonds represent blastocysts with presumable loss of a chromosome 15 from the sperm. White diamonds represent the methylation percentage for the ICRs on diploid chromosome 11. Average percent methylation for each cohort is indicated. E) Summary chart of percent methylation in all monosomy 11 blastocysts at the *SNRPN* (n = 5), *H19* (n = 25), and *KCNQ1OT1* (n = 25) ICRs. Black diamonds represent blastocysts with presumable loss of a chromosome 11 from the oocyte, grey diamonds represent blastocysts with presumable loss of a chromosome 11 from the sperm. White diamonds represent the methylation percentage for the ICR on diploid chromosome 15. The grey dotted box highlights the six blastocysts with unexpected *KCNQ1OT1* methylation profiles. Average percent methylation for each cohort is indicated. Methylation dot diagrams for individual monosomy blastocysts and diploid ICRs can be found in S3 and S4 Figs, and a summary can be found in S2 Table.

At imprinted regions, a monosomy chromosome results in a loss of either the inherited methylated allele or loss of the inherited unmethylated allele. Loss of the methylated allele is expected to result in hypomethylation of the ICR in the blastocyst, contributing only from the unmethylated chromosome. Loss of the unmethylated allele is expected to result in hypermethylation of the ICR in the blastocyst, contributing only from the methylated chromosome (Fig 3a–3c).

Analysis of monosomy 15 blastocysts at *SNRPN* revealed 15/20 blastocysts with an average methylation of 2.3% ± 1.0% (p<0.001 compared to controls), suggesting the chromosome loss originated from the oocyte, and 5/20 blastocysts with 87.8% ± 3.7% average methylation (p<0.001), suggesting the chromosome loss originated from the sperm (Fig 3d, S3 Fig, S2 Table). *H19* and *KCNQ1OT1* reside on diploid chromosome 11 in these blastocysts, and thus were unaffected by the monosomy in all five blastocysts examined, with an average methylation of 48.5% ± 5.4% (p = 0.64) and 52.0% ± 3.7% (p = 0.49), respectively (Fig 3d, S4 Fig, S2 Table).

Analysis of monosomy 11 blastocysts at *H19* revealed 19/25 blastocysts with an average methylation of 84.6% ± 6.4% (p<0.001), suggesting the chromosome loss arose from the oocyte, while 6/25 blastocysts had an average methylation of 4.2% ± 2.9% (p<0.001), suggesting the chromosome loss arose from the sperm (Fig 3e, S3 Fig, S2 Table). In the same monosomy 11 blastocysts, *KCNQ1OT1* had 16/25 blastocysts with 4.0% ± 1.6% (p<0.001) average methylation, suggesting the chromosome loss originated from the oocyte, and 3/25 blastocysts with 91.3% ± 6.4% (p<0.001) average methylation, suggesting the chromosome loss originated from the sperm (Fig 3e, S3 Fig, S2 Table). In confirmation of these results, both *H19* and *KCNQ1OT1* reflected the same origin of loss from the oocyte or sperm in the same individual blastocysts. For example, blastocysts 11M06, 11M10, 11M22 presumably lost the chromosome contributed from the sperm at both imprinted loci. A third subset of 6/25 blastocysts had 47.8% ± 6.9% (p = 0.25) average methylation for *KCNQ1OT1*, which was not statistically different from control blastocysts at this imprinted locus. *SNRPN* resides on diploid chromosome 15, and thus was unaffected by the monosomy 11 in all five blastocysts examined having 51.6% ± 2.9% (p = 0.29) average methylation (Fig 3e, S4 Fig, S2 Table).

### Imprinted gene expression in aneuploid blastocysts

**Fig 4:**
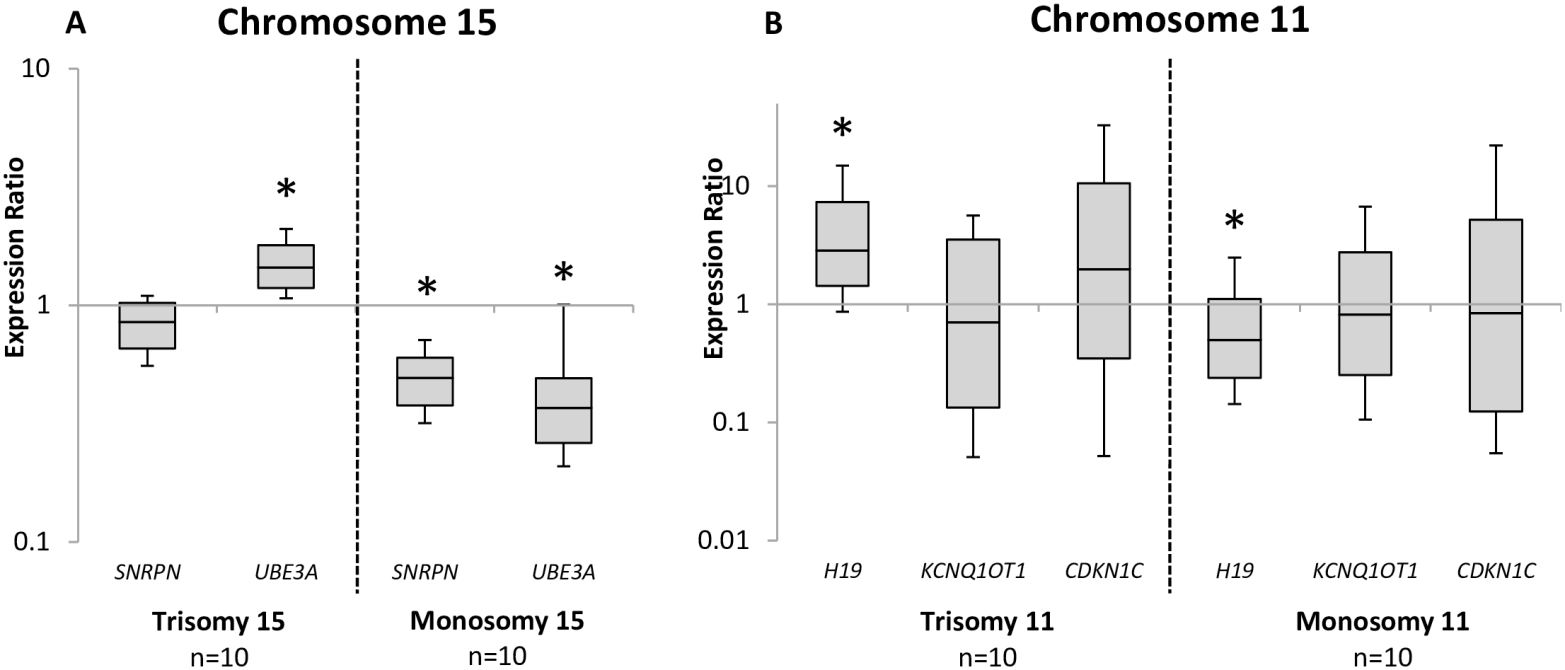
Summary of relative expression for imprinted genes on Chromosome 15 and Chromosome 11 in individual blastocysts. A) Relative gene expression of *SNRPN* and *UBE3A* in control blastocysts (n = 10) compared to trisomy 15 (n = 10) and monosomy 15 (n = 10) blastocysts. B) Relative gene expression of *H19*, *KCNQ1OT1* and *CDKN1C* in control blastocysts (n = 10) compared to trisomy 11 (n = 10) and monosomy 11 (n = 10) blastocysts. Relative expression between control and aneuploid groups was normalized to the housekeeping gene *PPIA* (7p13) using the Relative Expression Software Tool (REST) to determine an expression ratio that was tested for significance by a Pair Wise Fixed Reallocation Randomization Test. Differences were considered to be statistically significant when p<0.05, Star “*” denotes statistical significance.

Transcript abundance for trisomy 15 and monosomy 15 blastocysts were compared to control blastocysts at two imprinted genes on human chromosome 15, and normalized to the housekeeping gene, *PPIA* (7p13). *SNRPN* is paternally expressed, while *UBE3A* is maternally expressed in human blastocysts. *UBE3A* was significantly increased in the trisomy 15 blastocysts (p<0.001), while *SNRPN* was not statistically altered compared to controls (p = 0.08). In the monosomy 15 blastocysts, both *SNRPN* (p<0.001) and *UBE3A* (p<0.001) were significantly decreased when compared to the control blastocysts (Fig 4a).

In the same way, transcript abundance for trisomy 11 and monosomy 11 blastocysts were compared to control blastocysts at three imprinted genes on human chromosome 11, and normalized to the same housekeeping gene, *PPIA* (7p13). *H19* and *CDKN1C* are maternally expressed, while *KCNQ1OT1* is paternally expressed, in human blastocysts. *H19* (p<0.01) was significantly increased in the trisomy 11 blastocysts, while *CDKN1C* trended towards an increase without statistical difference (p = 0.30). *KCNQ1OT1* expression was comparable to controls (p = 0.49). In the monosomy 11 blastocysts, *H19* (p<0.05) was significantly decreased compared to the control group. While no significant difference was observed between the monosomy group and controls at *KCNQ1OT1* (p = 0.65) or *CDKN1C* (p = 0.79), a trend towards a decrease was evident (Fig 4b).

## Discussion

This study was designed to investigate the DNA methylation and imprinted gene expression profiles of imprinting control regions upon altered chromosome constitution in individual human blastocysts. Previously, our group examined the effects of aneuploidy on epigenetic modifications chromosome-wide. In monosomy blastocysts, DNA methylation levels were reduced on the chromosome involved in the error, while trisomy blastocysts showed no difference in DNA methylation compared to diploid controls. Likewise, protein-coding genes found on the specific monosomy chromosome were significantly decreased [24]. On account of these results, we questioned how whole chromosome aneuploidy can affect strict epigenetically regulated domains. Specifically, we questioned whether genomic imprints are maintained despite chromosome-wide reduced methylation levels on these monosomies. To investigate, we compared the DNA methylation profiles of trisomy 15 and 11, as well as monosomy 15 and 11, to control blastocysts. Three confirmed imprinting control regions shown to result in human imprinting disorders when dysregulated were selected for investigation; the *SNRPN* ICR, located on human chromosome 15, and the *H19* and *KCNQ1OT1* ICRs located on human chromosome 11.

### Trisomy blastocysts have altered imprinted DNA methylation

All forty trisomy blastocysts that were examined showed methylation profiles away from the expected fifty percent methylation for all imprinting control regions present on the aneuploid chromosome. When more than one single nucleotide polymorphism was evident at a gene within a blastocyst, both methylated and unmethylated DNA strands were often apparent under one SNP designation, highlighting the presence of three contributing chromosomes. Both *SNRPN* and *KCNQ1OT1* had predominantly increased methylation profiles compared to control blastocysts, while *H19* primarily had decreased methylation compared to controls. The *SNRPN* and *KCNQ1OT1* ICRs establish methylation in the oocyte and are unmethylated in the sperm, thus we can presume that the third additional chromosome was gained from the oocyte due to meiotic errors for the majority of blastocysts (20/20 *SNRPN*, 17/20 *KCNQ1OT1*). Likewise, as the *H19* ICR has the reverse imprint, establishing methylation in the sperm and remaining unmethylated in the oocyte, we can again presume that the third additional chromosome was gained primarily from the oocyte (17/20 *H19*). In support of our results, both *H19* and *KCNQ1OT1*, located contiguously on chromosome 11, reflect the same gain from either the oocyte or sperm in the same individual blastocysts. Maternal meiotic errors reportedly do not affect all chromosomes equally, with elevated rates for some chromosomes (including trisomy 15) [4], providing explanation for the observed 100% presumably maternal gain in this cohort of trisomy 15 blastocysts but not in this cohort of trisomy 11 blastocysts.

Importantly, imprinted methylation is altered by the trisomy in all forty aneuploid blastocysts examined, having both increased and decreased methylation detected depending on the ICR and the origin of the third chromosome. The imprinted regions present on diploid chromosomes were unaffected by the presence of the trisomic chromosome and displayed methylation profiles comparable to controls, suggesting that the altered imprinted methylation is restricted to the chromosome involved in the trisomy.

### Monosomy blastocysts have altered imprinted DNA methylation

Similar to the trisomy blastocysts, monosomy blastocysts appeared to bias towards presumptive chromosomal loss from the oocyte, likely a consequence of oocyte meiotic errors (15/20 *SNRPN*, 19/25 *H19*, 16/25 *KCNQ1OT1*). In support of these observations, both *H19* and *KCNQ1OT1* again reflected the same loss from the oocyte or sperm in the same individual blastocysts. Meiotic errors in sperm are rare, with only ~5% of sperm identified as aneuploid [34], and paternal error is extremely low in blastocyst biopsies, having a higher frequency in monosomies than in trisomies [4]. In total, 7.5% of the trisomy blastocysts and 24.4% of the monosomy blastocysts examined in this cohort were presumably paternal meiotic in origin.

To summarize, all forty-five monosomy blastocysts that were examined showed altered methylation away from the expected fifty percent methylation in at least one imprinting control region. Unexpectedly, a third subset of imprinted DNA methylation was observed for the *KCNQ1OT1* ICR. In this group, 6/25 blastocysts more closely resembled the methylation profile of controls, while *H19* remained altered in the same individual blastocysts. This suggests that these unpredicted results are restricted to the *KCNQ1OT1* domain. Importantly, due to the monoallelic nature of imprints, some monosomy blastocysts appeared to gain imprinted methylation in at least one ICR compared to controls (for example, 19/25 at *H19*) due to the origin of the single remaining chromosome. Similar to the trisomies examined, imprinted regions present on diploid chromosomes were unaffected by the monosomy and displayed expected methylation profiles, suggesting that the altered methylation is again restricted to the single remaining chromosome involved in the monosomy.

### Altered imprinted gene expression in aneuploid blastocysts

Both maternally and paternally expressed genes can reside within imprinted regions, regardless of the origin of ICR methylation. Thus, we further investigated the impact of this observed altered imprinted DNA methylation found in aneuploid blastocysts on the expression of the imprinted genes that exist within the regions.

On human chromosome 15, *SNRPN* is a paternally expressed gene, and *UBE3A* is a maternally expressed gene under control of the same ICR. A significant increase in *UBE3A* was observed in trisomy 15 blastocysts, while *SNRPN* was unaffected compared to controls. All 100% of the trisomy 15 blastocysts examined for imprinted methylation presumably gained an additional methylated maternal chromosome. This third chromosome arising from the oocyte likely increased maternal *UBE3A* expression, while *SNRPN* expression from the paternal contribution remained unaffected.

In the monosomy 15 blastocysts, both *SNRPN* and *UBE3A* were significantly decreased in expression when compared to the control blastocysts. A loss of the oocyte-derived chromosome presumably occurred in 75% of blastocysts, resulting in loss of *UBE3A* expression. The other 25% of blastocysts had presumably lost the sperm-derived chromosome, resulting in loss of *SNRPN* expression. The remaining allele in both scenarios likely was unaffected. Thus, both genes appear decreased in comparison to diploid controls.

On human chromosome 11, *KCNQ1OT1* is a paternally expressed non-coding RNA, while *CDKN1C* is a maternally expression gene within the same ICR. *H19* is a maternally expressed non-coding RNA located at an adjacent ICR. A significant increase in maternally-expressed *H19* was observed in trisomy 11 blastocysts, while a trend towards an increase was also observed for *CDKN1C*. *KCNQ1OT1* was not statistically different from controls. With respect to methylation, 85% of trisomy 11 blastocysts examined for imprinted DNA methylation presumably originated from an additional methylated maternal chromosome, increasing the maternal contribution for *H19* and *CDKN1C* expression, while *KCNQ1OT1* expression from the paternal contribution remained unaffected.

In the monosomy 11 blastocysts, a significant decrease in *H19* was observed, while both *KCNQ1OT1* and *CDKN1C* only trended towards a decrease when compared to the control blastocysts. In 76% of monosomy 11 blastocysts examined for imprinted methylation, the oocyte-derived chromosome was presumably lost, resulting in loss of *H19 and CDKN1C* expression, while the other 24% had presumably lost the sperm-derived chromosome, resulting in loss of *KCNQ1OT1* expression. Again, the remaining allele likely was unaffected in both scenarios. Thus, all three genes appear decreased in comparison to controls, with a statistical significance observed at *H19*.

Overall, the imprinted gene expression results correlated with our expectations from the observed imprinted DNA methylation results in their respective aneuploid blastocysts. Interestingly, the genes present within the *KCNQ1OT1* ICR had more variable expression and did not show statistically significant differences compared to controls. These expression results may further support the concept that the *KCNQ1OT1* region behaves differently than other ICRs at the human blastocyst stage.

All four aneuploid blastocyst groups, independent of the chromosome involved in the error, had significantly altered gene expression in at least one gene, reinforcing the notion that aneuploid blastocysts are not developmentally competent. As imprinted genes are critical for embryonic development, this disrupted imprinted expression likely contributes to the frequent implantation failure observed from aneuploid embryos.

### Aneuploidy and other chromosome errors at imprinting control regions

Human imprinting disorders are a consequence of chromosomal mutations, microdeletions or microduplications within the imprinted regions [35], imprinted methylation defects, and uniparental disomy (UPD) [19, 36, 37]. UPD is classified as an inheritance of two chromosome copies (or chromosome regions) from one parent and no copy from the other parent [38]. These occurrences are generally undetectable in common embryo chromosome screening techniques like array CGH or qPCR on account of normal diploid copy numbers, yet the parental-derived imprinted DNA methylation profile is dramatically affected. Likewise, mutations and microdeletions or microduplications of imprinted regions often go undetected based on the limited region of chromosome affected. The disrupted DNA methylation at these ICRs, however, and resulting aberrant gene expression of the underlying imprinted genes can lead to imprinting disorders, like Beckwith-Wiedemann Syndrome [19, 39], Angelman Syndrome and Prader-Willi Syndrome [22, 37].

A publication using SNP array-based 24-chromosome aneuploidy screening reported that the frequency of UPD in human blastocysts is extremely rare (0.06%) [40]. By comparison, full chromosome aneuploidy of oocytes and preimplantation embryos is exceptionally common in human reproduction [41–48]. In fact, it is estimated that over half of early human IVF embryos are affected by whole-chromosome imbalances [4, 48]. Maternal meiotic errors originating in the oocyte are responsible for the vast majority of these imbalances [3, 4], while the frequency of mitotic errors and the level of embryo mosaicism are still speculative. A recent report from a preimplantation genetic diagnosis (PGD) commercial laboratory observed 30% of blastocysts to be mosaic from a multitude of IVF clinics worldwide [49]. The authors go on to emphasize that blastocyst mosaicism rates vary between IVF clinics, pointing at possible procedure-related effects on post-meiotic chromosome segregation errors. Our in-house genetics laboratory at the Colorado Center for Reproductive Medicine routinely reconfirms diagnosis on aneuploid embryos. A dual trophectoderm biopsy is performed with blinded analysis of prior comprehensive chromosome screening (CCS) aneuploidy results to reveal 96% concordance within an individual blastocyst and only a 4% false-positive rate of mosaic aneuploid embryos.

Our study was the first to investigate if whole-chromosome imbalances impact these imprinting control regions differently than microdeletions, UPDs, or methylation defects restricted to individual ICRs. In fact, this study is also the first to identify an imprinted region, *KCNQ1OT1*, that responds differently to a single whole-chromosome monosomy state in a subset of human blastocysts. Investigations into the rationale for these observations are ongoing. There is a possibility that it could reflect the position of the ICR within the domain, the DNA sequence itself, a combination of both, or some other mechanism in which we are currently unaware. Intriguingly, unlike the *SNRPN* and *H19* ICRs, the *KCNQ1OT1* ICR resides directly inside the coding region of the gene *KCNQ1*. This could create a more susceptible chromatin conformation that would enable targeting of DNA methyltransferase (DNMT) enzymes or tet methylcytosine dioxygenase (TET) machinery resulting in DNA methylation acquisition or loss, respectively, in these monosomy blastocysts. There is also potential that this region has a relaxed imprinted regulation compared to other ICRs at this stage in human development.

### Genomic imprinting in human blastocysts

Limited studies have examined genomic imprinting in human blastocysts, focused particularly in relation to the effects of ART treatments (reviewed in [50]). Principally, these studies were performed on poor quality embryos that were not suitable for transfer [51–54], however good quality blastocysts have also revealed an unusually high frequency of aberrant methylation patterns [27, 29, 30]. At the blastocyst stage, 8/12 (67%) high quality transferable blastocysts harbored aberrant methylation for *SNRPN* [27], while 2/5 (40%) [29], and 4/13 (31%) [27], blastocysts displayed aberrant methylation for *KCNQ1OT1*, and 2/14 (14%) blastocysts harboured aberrant *H19* methylation [27]. Conversely, two studies both showed normal *H19* methylation in five blastocysts, respectively [29, 30]. Discrepancies between studies may relate to the small number of embryos analyzed. Notably, while SNP analysis was possible for a select few, comprehensive chromosome screening was not performed on these embryos prior to these methylation studies.

In contrast, our study which controls for chromosome constitution, suggests that diploid blastocysts harbor imprinted methylation profiles within the expected normal range, while trisomy and monosomy blastocysts have altered methylation at these imprinted loci. Combined with inherent underlying infertility, this provides a possibility that human blastocyst methylation errors are more closely linked to chromosomal aneuploidy than the infertility treatments themselves.

## Conclusions

All trisomy blastocysts examined showed a methylation profile consistent with a third inherited chromosome for at least one ICR, presumably originating in the oocyte for the majority of the blastocysts. This supports known literature that maternal meiotic errors are the most common cause of trisomies. Significantly increased and decreased ICR methylation compared to controls contributed to altered imprinted gene expression. All imprinted genes examined in trisomy blastocysts had either significantly increased expression or comparable expression to controls, providing one explanation as to why trisomy embryos have the ability to continue into post-implantation development, but mostly fail to develop to term.

All monosomy blastocysts examined showed a methylation profile consistent with a loss of an inherited chromosome. This chromosome loss is also presumably originating in the oocyte for the majority of the blastocysts, emphasizing that maternal meiotic errors are also the most common cause of monosomies. Significant hypermethylated and hypomethylated ICRs compared to controls contributed to altered imprinted gene expression. By contrast to their trisomy counterparts, all monosomy blastocysts exhibited significantly decreased expression in at least one imprinted gene, with a downward trend observed in all imprinted genes analyzed. This loss of imprinted gene expression may equally contribute to an explanation as to why monosomy embryos cannot progress further than the preimplantation period of development, contributing to their compromised developmental potential.

## Supporting Information

S1 Fig Imprinted DNA methylation profiles of individual control blastocysts. Imprinted DNA methylation analysis in twenty individual control blastocysts at the A) *SNRPN* ICR, B) *H19* ICR, C) *KCNQ1OT1* ICR. Each line is a unique DNA strand amplified within an individual blastocyst. Black dots represent methylated CpGs, white dots represent unmethylated CpGs. Blastocyst ID is indicated above-left of the diagram, in which “C##” represents control blastocyst number. SNP ID and percent methylation is indicated above-right of the diagram. When more than one SNP is present, a SNP ID and percent methylation is indicated separately for each SNP.
(TIF)

S2 Fig Imprinted DNA methylation profiles of individual trisomy blastocysts. Imprinted DNA methylation analysis in twenty individual trisomy 15 blastocysts at the A) *SNRPN* ICR; and twenty individual trisomy 11 blastocysts at the B) *H19* ICR, and C) *KCNQ1OT1* ICR. Each line is a unique DNA strand amplified within an individual blastocyst. Black dots represent methylated CpGs, white dots represent unmethylated CpGs. Blastocyst ID is indicated above-left of the diagram, in which “15T##” represents trisomy 15 blastocyst number, and “11T##” represents trisomy 11 blastocyst number. SNP ID and percent methylation is indicated above-right of the diagram. When more than one SNP is present, a SNP ID and percent methylation is indicated separately for each SNP. Blastocysts with ID and percent methylation denoted in grey are presumable chromosome gain from the sperm.
(TIF)

S3 Fig Imprinted DNA methylation profiles of individual monosomy blastocysts. Imprinted DNA methylation analysis in twenty individual monosomy 15 blastocysts at the A) *SNRPN* ICR; and twenty-five individual monosomy 11 blastocysts at the B) *H19* ICR, and C) *KCNQ1OT1* ICR. Each line is a unique DNA strand amplified within an individual blastocyst. Black dots represent methylated CpGs, white dots represent unmethylated CpGs. Blastocyst ID is indicated above-left of the diagram, in which “15M##” represents monosomy 15 blastocyst number, and “11M##” represents monosomy 11 blastocyst number. SNP ID and percent methylation is indicated above-right of the diagram. When more than one SNP is present, a SNP ID and percent methylation is indicated separately for each SNP. Blastocysts with ID and percent methylation denoted in grey are presumable chromosome loss from the sperm. The grey boxes highlight the six blastocysts with *KCNQ1OT1* methylation profiles comparable to controls.
(TIF)

S4 Fig Diploid imprinted DNA methylation profiles of individual aneuploid blastocysts. Diploid imprinted DNA methylation analysis in five trisomy 11 and five monosomy 11 blastocysts at the A) *SNRPN* ICR, and five trisomy 15 and five monosomy 15 blastocysts at the B) *H19* ICR, C) *KCNQ1OT1* ICR. Each line is a unique DNA strand amplified within an individual blastocyst. Black dots represent methylated CpGs, white dots represent unmethylated CpGs. Blastocyst ID is indicated above-left of the diagram, in which “15T##” represents trisomy 15 blastocyst number, “15M##” represents monosomy 15 blastocyst number, “11T##” represents trisomy 11 blastocyst number, and “11M##” represents monosomy 11 blastocyst number. SNP ID and percent methylation is indicated above-right of the diagram. When more than one SNP is present, a SNP ID and percent methylation is indicated separately for each SNP.
(TIF)

S1 Table SNP frequencies among blastocyst groups. A single nucleotide polymorphism (SNP) was present in all three amplified regions, but at different frequencies: *SNRPN* SNP G (84.8%) / A (15.2%) (Rs220029), *H19* SNP C (66.4%) / A (33.6%) (Rs2071094), and *KCNQ1OT1* SNP G (94.7%) / A (6.3%) (Rs56134313).
(TIF)

S2 Table Methylation results per blastocyst. An overview table indicating DNA methylation percentages for all relevant ICRs per blastocyst. C## = Control blastocyst, 15T## = Trisomy 15 blastocyst, 11T## = Trisomy 11 blastocyst, 15M## = Monosomy 15 blastocyst, 11M## = Monosomy 11 blastocyst, Grade = numeric:alpha:alpha score for blastocyst degree of expansion and hatching status: ICM development: TE development [25], Vit = vitrification freezing, SNP = single nucleotide polymorphism, light grey sections = presumable paternal gain or loss originating from the sperm, dark grey sections = unexpected subset of methylation profiles at *KCNQ1OT1*.
(TIF)
