# Supplementary figures and images for: Epigenetic Dysregulation Observed in Monosomy Blastocysts Further Compromises Developmental Potential
Source: PLoS One. 2016 Jun 7;11(6):e0156980. doi: 10.1371/journal.pone.0156980 (PMC4896457; doi:10.1371/journal.pone.0156980)

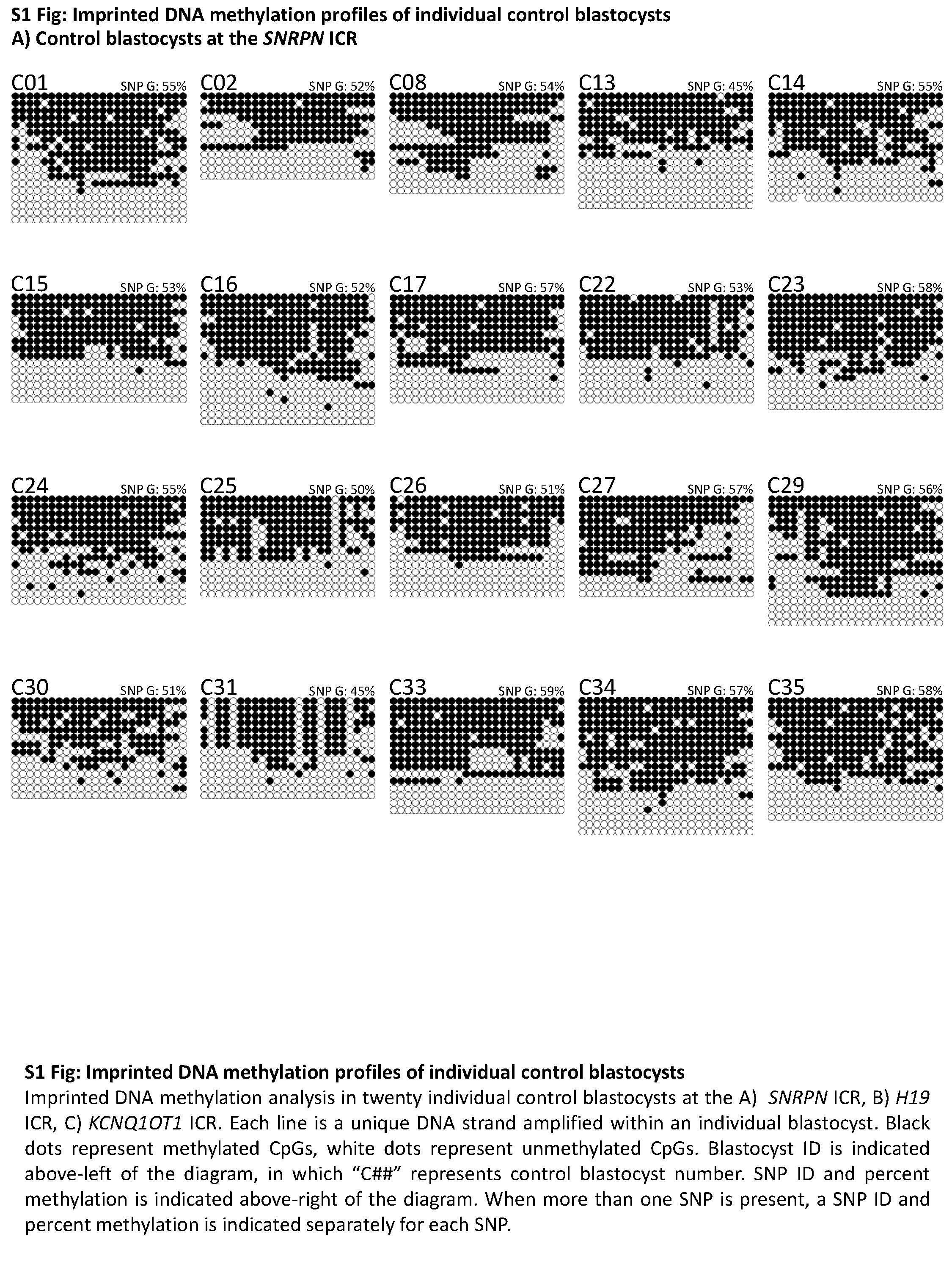

Supplement: S1 Fig — Imprinted DNA methylation analysis in twenty individual control blastocysts at the A) SNRPN ICR, B) H19 ICR, C) KCNQ1OT1 ICR. Each line is a unique DNA strand amplified within an individual blastocyst. Black dots represent methylated CpGs, white dots represent unmethylated CpGs. Blastocyst ID is indicated above-left of the diagram, in which “C##” represents control blastocyst number. SNP ID and percent methylation is indicated above-right of the diagram. When more than one SNP is present, a SNP ID and percent methylation is indicated separately for each SNP. (TIF) [file pone.0156980.s001.tif]

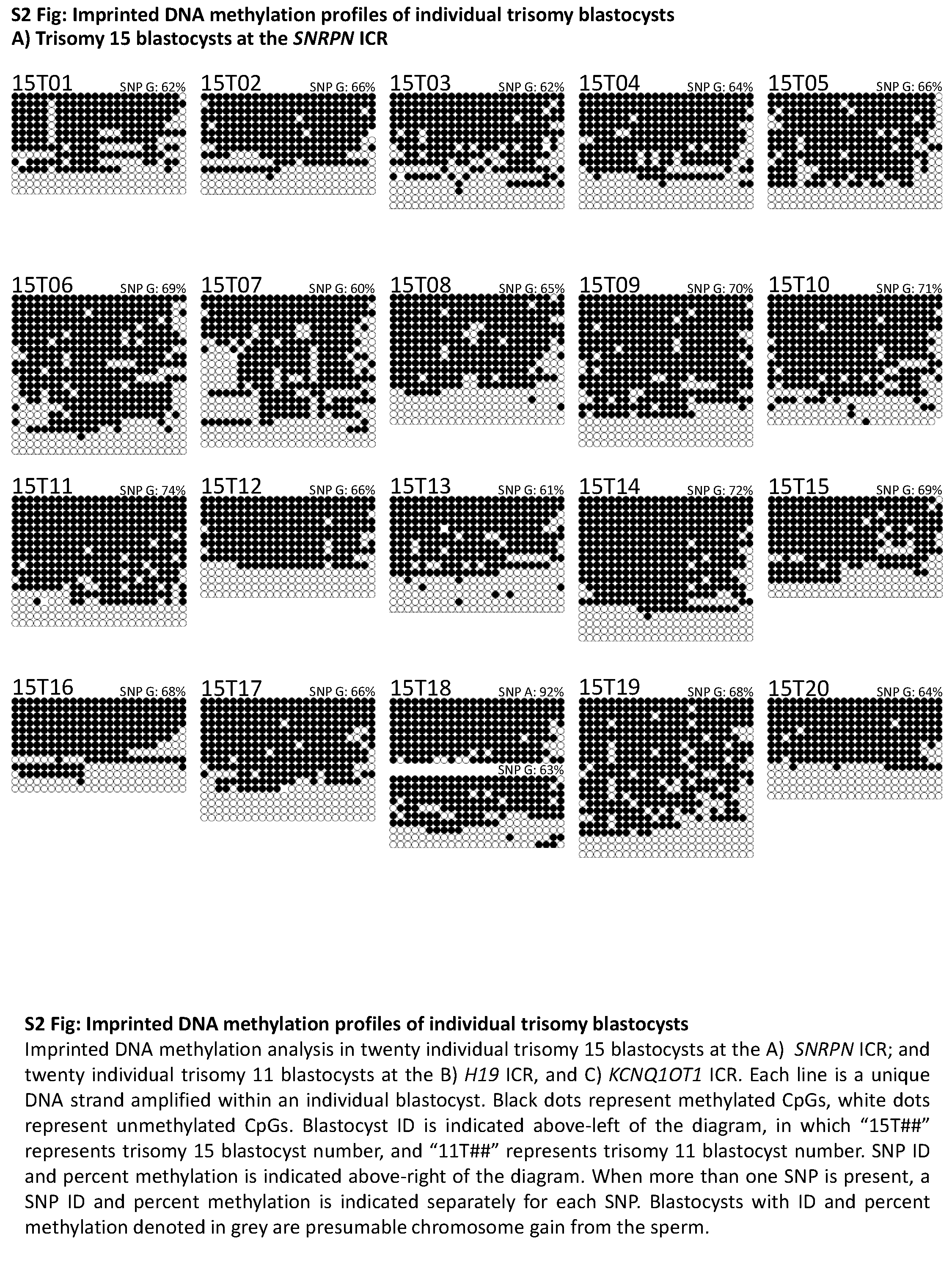

Supplement: S2 Fig — Imprinted DNA methylation analysis in twenty individual trisomy 15 blastocysts at the A) SNRPN ICR; and twenty individual trisomy 11 blastocysts at the B) H19 ICR, and C) KCNQ1OT1 ICR. Each line is a unique DNA strand amplified within an individual blastocyst. Black dots represent methylated CpGs, white dots represent unmethylated CpGs. Blastocyst ID is indicated above-left of the diagram, in which “15T##” represents trisomy 15 blastocyst number, and “11T##” represents trisomy 11 blastocyst number. SNP ID and percent methylation is indicated above-right of the diagram. When more than one SNP is present, a SNP ID and percent methylation is indicated separately for each SNP. Blastocysts with ID and percent methylation denoted in grey are presumable chromosome gain from the sperm. (TIF) [file pone.0156980.s002.tif]

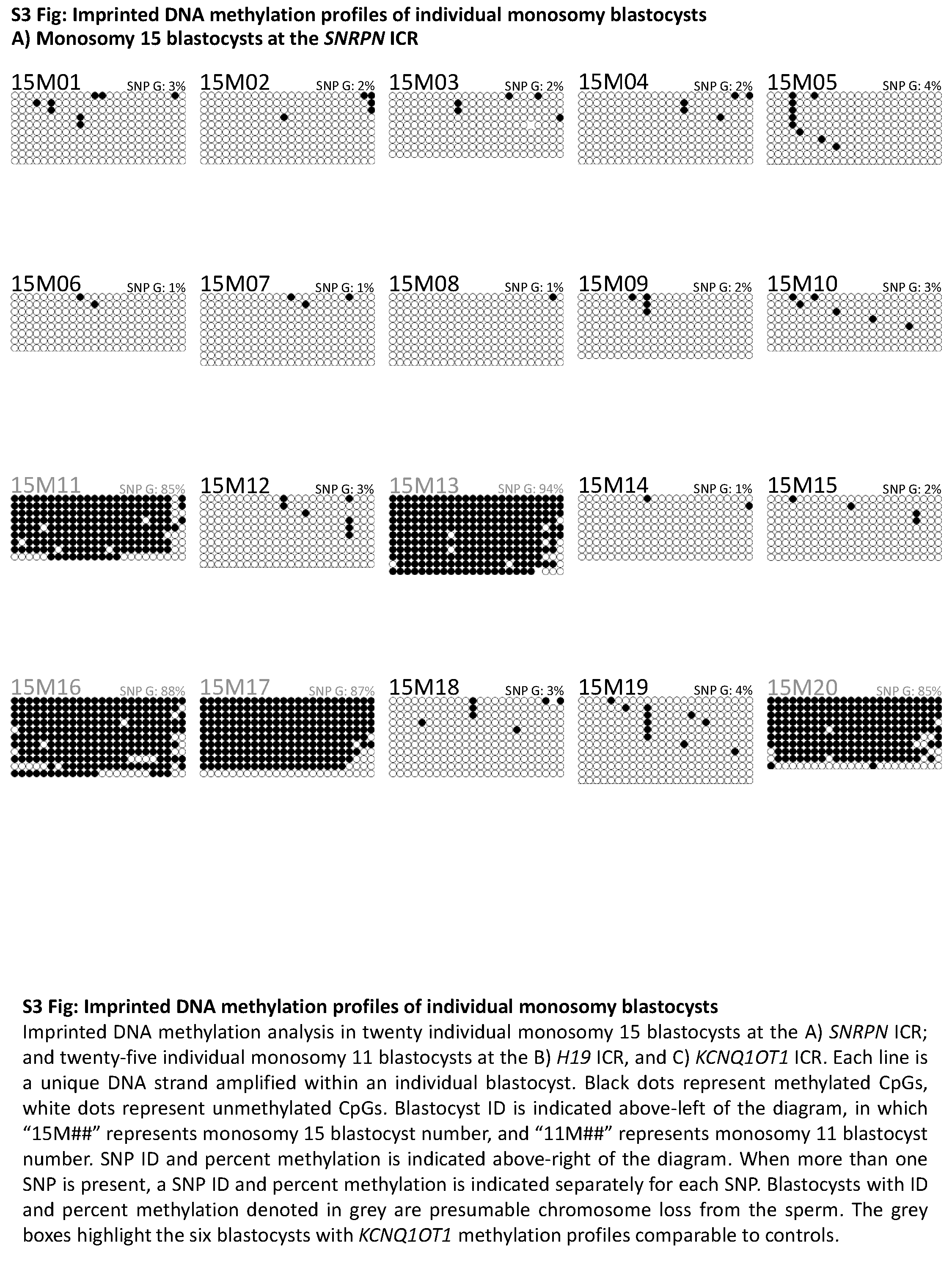

Supplement: S3 Fig — Imprinted DNA methylation analysis in twenty individual monosomy 15 blastocysts at the A) SNRPN ICR; and twenty-five individual monosomy 11 blastocysts at the B) H19 ICR, and C) KCNQ1OT1 ICR. Each line is a unique DNA strand amplified within an individual blastocyst. Black dots represent methylated CpGs, white dots represent unmethylated CpGs. Blastocyst ID is indicated above-left of the diagram, in which “15M##” represents monosomy 15 blastocyst number, and “11M##” represents monosomy 11 blastocyst number. SNP ID and percent methylation is indicated above-right of the diagram. When more than one SNP is present, a SNP ID and percent methylation is indicated separately for each SNP. Blastocysts with ID and percent methylation denoted in grey are presumable chromosome loss from the sperm. The grey boxes highlight the six blastocysts with KCNQ1OT1 methylation profiles comparable to controls. (TIF) [file pone.0156980.s003.tif]

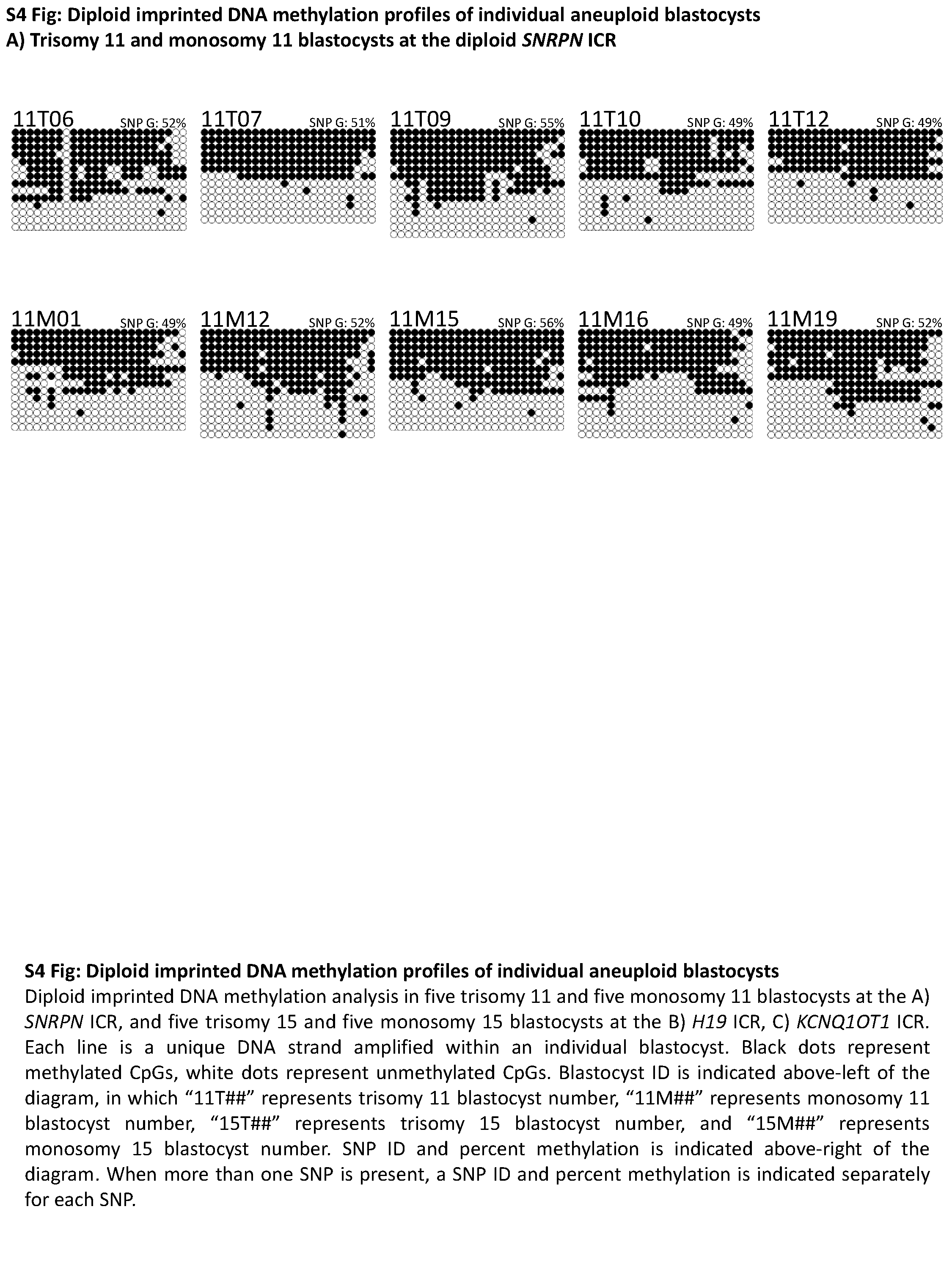

Supplement: S4 Fig — Diploid imprinted DNA methylation analysis in five trisomy 11 and five monosomy 11 blastocysts at the A) SNRPN ICR, and five trisomy 15 and five monosomy 15 blastocysts at the B) H19 ICR, C) KCNQ1OT1 ICR. Each line is a unique DNA strand amplified within an individual blastocyst. Black dots represent methylated CpGs, white dots represent unmethylated CpGs. Blastocyst ID is indicated above-left of the diagram, in which “15T##” represents trisomy 15 blastocyst number, “15M##” represents monosomy 15 blastocyst number, “11T##” represents trisomy 11 blastocyst number, and “11M##” represents monosomy 11 blastocyst number. SNP ID and percent methylation is indicated above-right of the diagram. When more than one SNP is present, a SNP ID and percent methylation is indicated separately for each SNP. (TIF) [file pone.0156980.s004.tif]

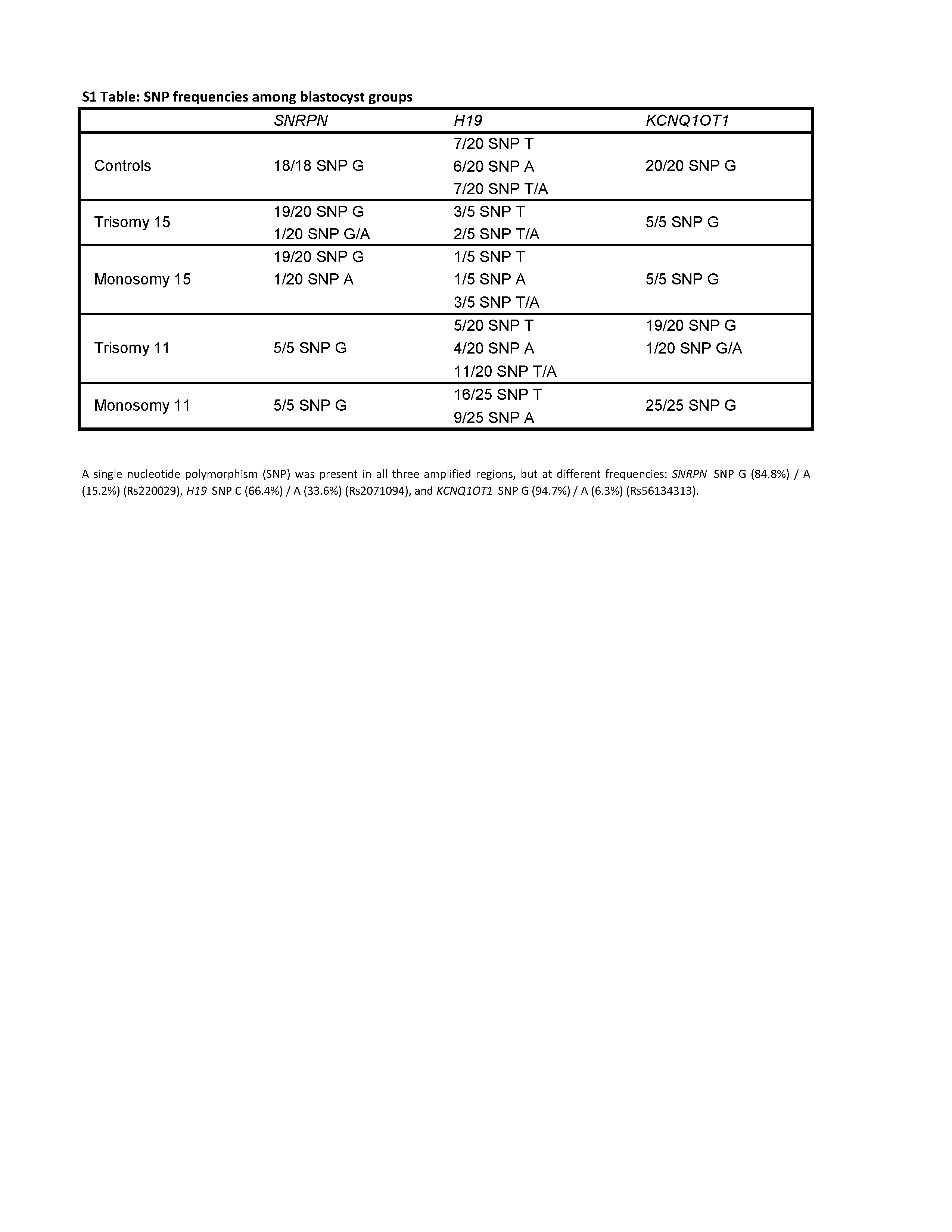

Supplement: S1 Table — A single nucleotide polymorphism (SNP) was present in all three amplified regions, but at different frequencies: SNRPN SNP G (84.8%) / A (15.2%) (Rs220029), H19 SNP C (66.4%) / A (33.6%) (Rs2071094), and KCNQ1OT1 SNP G (94.7%) / A (6.3%) (Rs56134313). (TIF) [file pone.0156980.s005.tif]

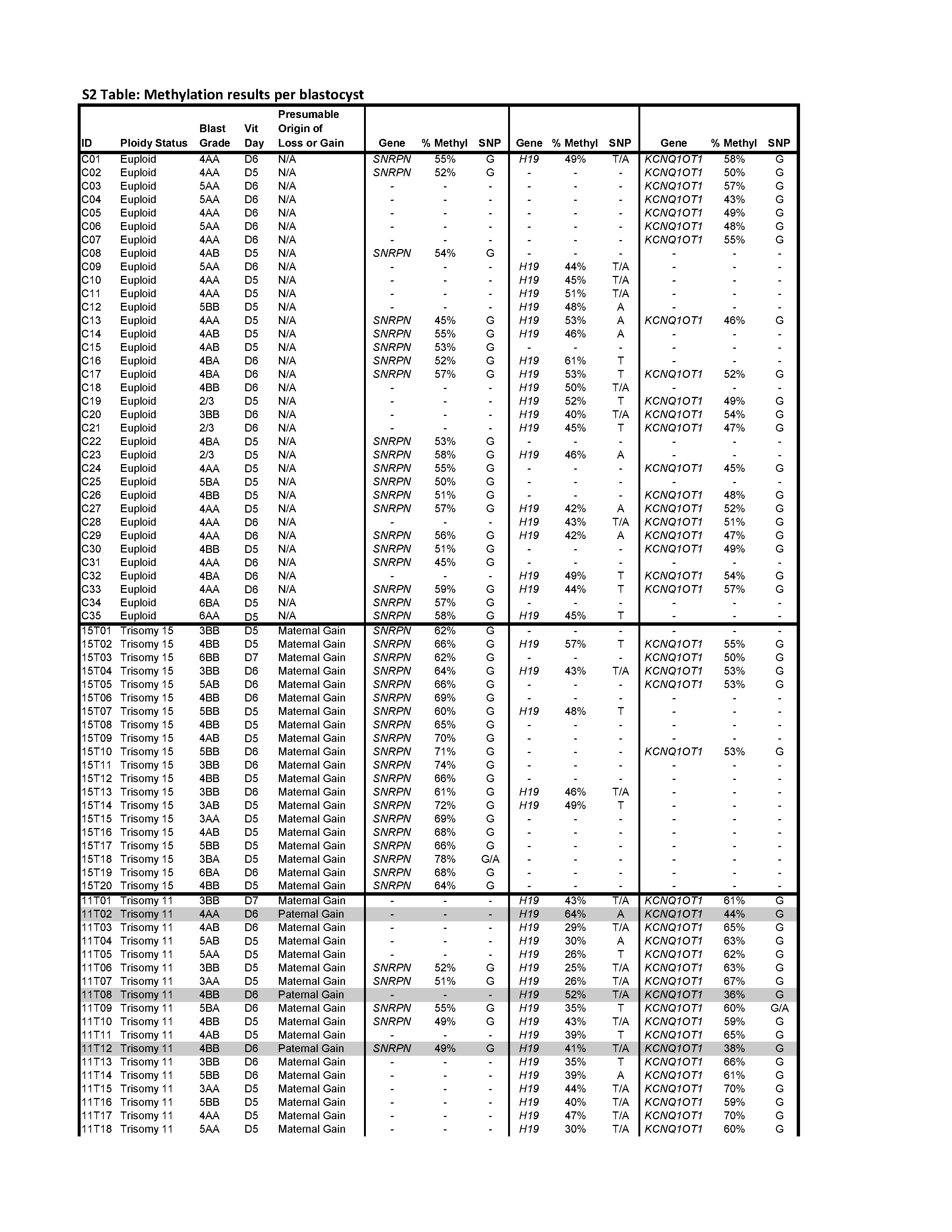

Supplement: S2 Table — An overview table indicating DNA methylation percentages for all relevant ICRs per blastocyst. C## = Control blastocyst, 15T## = Trisomy 15 blastocyst, 11T## = Trisomy 11 blastocyst, 15M## = Monosomy 15 blastocyst, 11M## = Monosomy 11 blastocyst, Grade = numeric:alpha:alpha score for blastocyst degree of expansion and hatching status: ICM development: TE development [25], Vit = vitrification freezing, SNP = single nucleotide polymorphism, light grey sections = presumable paternal gain or loss originating from the sperm, dark grey sections = unexpected subset of methylation profiles at KCNQ1OT1. (TIF) [file pone.0156980.s006.tif]
